# Allergic Diseases and Risk of Malignancy of Gastrointestinal Cancers

**DOI:** 10.3390/cancers15123219

**Published:** 2023-06-16

**Authors:** Yoon Jin Choi, Kyungdo Han, Eun Hyo Jin, Joo Hyun Lim, Cheol Min Shin, Dong Ho Lee

**Affiliations:** 1 Department of Gastroenterology, National Cancer Center, Goyang-si 10408, Republic of Korea; yjinchoi@yuhs.ac; 2 Department of Statistics and Actuarial Science, Soongsil University, Seoul 06978, Republic of Korea; 3 Department of Internal Medicine, Healthcare Research Institute, Seoul National University Hospital Healthcare System Gangnam Center, Seoul 06236, Republic of Korea; 65845@snuh.org (E.H.J.); limz00@gmail.com (J.H.L.); 4 Department of Internal Medicine, Seoul National University Bundang Hospital, Seongnam-si 13620, Republic of Korea; scm6md@gmail.com; 5 Department of Internal Medicine, Liver Research Institute, Seoul National University College of Medicine, Seoul 03080, Republic of Korea

**Keywords:** allergy, cancer epidemiology, gastrointestinal cancer

## Abstract

**Simple Summary:**

It is thought that allergic diseases could hamper or trigger carcinogenesis by enhancing host immune defenses or persisting inflammation, respectively. The gastrointestinal (GI) tract plays a crucial role in building the immune system via the gut barrier and through communicating with the microbiome. We hypothesized that allergic diseases may be linked with the occurrence of GI tract cancers. Analyzing national health check-up data, we found that, overall, allergic diseases which consist of allergic rhinitis, asthma, and atopic dermatitis reduced the risk of malignancy in the esophagus, stomach, colorectum, and liver. Given that gastric and liver cancers are typical infection-related malignancies, the inverse associations between allergic diseases and the subsequent development of GI cancers in the present study supports the cancer immunosurveillance hypothesis. This result could provide a clue for the novel approach to the prevention and treatment of GI cancers.

**Abstract:**

The aim of this study was to investigate the effect of allergic diseases, including allergic rhinitis, asthma, and atopic dermatitis, on the development of gastrointestinal (GI) cancers. We analyzed 9,892,633 Korean adults who underwent a medical check-up in the year 2009, and they were followed up until the year 2017. Allergic diseases and cancers were defined using the International Classification of Disease Codes. A Cox proportional hazards model was adapted to calculate the hazard ratios (HRs) and 95% confidence intervals (CIs). During a 7.3-year follow-up period, 48,045 patients were diagnosed with cancer. For all-combined allergic diseases, significant inverse associations were observed for cancers of the esophagus, stomach, colorectum, and liver (adjusted hazard ratios (aHRs [95% confidence interval, CI] 0.86 [0.82–0.91], 0.93 [0.91–0.94], 0.95 [0.93–0.96], and 0.90 [0.88–0.92], respectively). The sex-stratified analysis showed that the preventive effect of allergic diseases was persistent in gastric, colorectal, and liver cancers regardless of sex, while the inverse associations with esophageal and pancreatic cancers were observed only in men (aHR [95% CI] 0.84 [0.80–0.89] and 0.96 [0.93–0.99]). Allergic diseases, particularly allergic rhinitis, in adults were significantly associated with a decreased risk of most GI cancers, except for gallbladder and biliary tract cancers.

## 1. Introduction

Allergic diseases are a group of immune-mediated disorders mainly caused by an immunoglobulin (Ig)E-dependent immunological reaction to a harmless environmental antigen. These diseases include allergic dermatitis (AD), asthma, and allergic rhinitis (AR). The prevalence of allergic diseases has increased over the past decades, reaching about 20% worldwide [1,2].

Several epidemiologic studies have reported that asthma, allergies, or increased serum IgE levels show an inverse association with the risk of various cancers [3,4,5,6,7,8,9,10,11,12,13,14,15,16,17,18,19]. This is in line with the principle of immune checkpoint inhibitors, which bind to molecules on immune cells and reinforce human immune defences to eliminate cancer cells. However, these studies have shown inconsistent results, and some research suggests that allergic diseases may increase the risk of cancer by triggering persistent inflammation [20,21,22]. Possible reasons for the inconsistent results include a small sample size or different definitions of allergic diseases.

The gastrointestinal tract is a major organ constituting the immune system [23]. Atopic predisposition starts from the altered normal barrier function. Which means that disrupted skin or intestinal mucosa cannot protect the body anymore from the external environment, resulting in an abnormal immune response to ubiquitous antigens [24]. The human immune system also plays an important role in carcinogenesis, and immunotherapy has recently developed as an emerging part of cancer treatment [25,26]. In this context, allergic diseases could affect the occurrence of GI tract cancers, but there are not many studies focusing on the association between GI cancers and allergic diseases.

We aimed to evaluate the association between GI cancers and the allergic disease group consisting of AD, AR, and asthma (or the individual diseases), using a national database. We also assessed whether gender could influence the association.

## 2. Materials and Methods

### 2.1. Data Source

We used the Korean National Health Insurance Services (NHIS) database for this study. NHIS, the single government insurer in South Korea, runs mandatory social health insurance. The database consists of age, sex, monthly insurance premium, place of residence, and medical claims (disease codes, procedures, and prescriptions). Health examination data (height, weight, waist circumference, blood pressure measured by trained medical personnel, and serum glucose, total cholesterol, HDL cholesterol, LDL cholesterol, and triglyceride levels measured from at least 8 h-fasted blood) and a lifestyle questionnaire (smoking and alcohol consumption status, and exercise) were also included.

All procedures involving human participants were performed in accordance with the ethical standards of the Institutional and National Research Committees and the 1964 Helsinki declaration, including its later amendments or comparable ethical standards.

The Institutional Review Board of Seoul National University Bundang Hospital approved this study (X-2006-618-901). The requirement for informed consent was waived because the study was based on routinely collected insurance data. All authors had access to the study data and reviewed and confirmed the final manuscript.

### 2.2. Study Population and Data Collection

**Figure 1:**
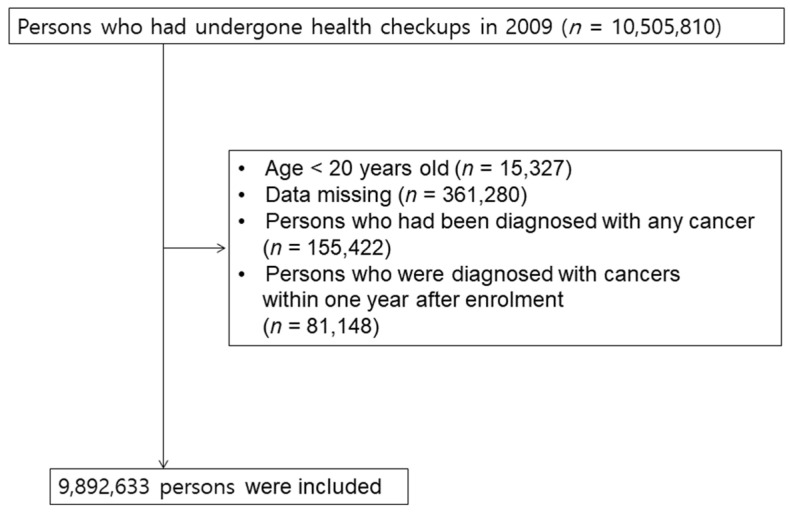
Flow chart for study population.

We recruited 10,490,483 subjects who were older than 20 years and who underwent an initial baseline health check-up by the NHIS in 2009. This cohort was followed up until 31 December 2017. From this initial population, we excluded 361,280 subjects who had missing data and 155,422 subjects who were diagnosed with any type of cancer during the preceding years. To clarify the temporal relationship, 81,148 patients who were diagnosed with cancers within one year after enrolment were excluded (lag period) (Figure 1).

Smoking status was divided into three stages as follows: never, former, and current. Regular exercise was defined as moderate physical activity at least 30 min per week and at least 5 days per week for the past week. Hypertension, diabetes mellitus, and dyslipidaemia were determined based on the physicians’ diagnoses (ICD-codes) or medication. Household income status was classified into 4 groups by quartile.

### 2.3. Disease Identification and Study Outcomes

Patients with the first diagnosis of allergic rhinitis (AR; ICD-10 J301–J304), allergic dermatitis (AD; ICD-10 L20), and asthma (ICD-10 J45–J46) from 1 January 2009 to 31 December 2009 were identified and comprised the cohort with allergic diseases. To increase the accuracy of the operational definition using ICD, only those who were diagnosed with an allergic disease at least three times per year were defined as having true allergy [27,28]. People who were never diagnosed with an allergic disease during the same follow-up period were designated as controls.

The primary endpoint was newly diagnosed esophagus, stomach, colorectal, gallbladder and biliary tract, pancreas and liver cancer, defined as new claims for inpatient or outpatient care with the diagnosis code of C10 (by C15 malignant neoplasm of the esophagus, C16 stomach, C18 colon, C19, C20 rectum, C24 biliary tract, C25 pancreas and C22 liver) code with registration in the special co-payment reduction programme for critical illness, which requires a certification for the physicians. The cohort was followed from the date of the health check-up to the date of the incident gastric, colorectal, and liver cancer, death, or until the end of the study period (31 December 2017).

### 2.4. Statistical Analysis

The basic characteristics of the study population are presented by descriptive statistics. Cox regression analysis, adjusted for age, sex, BMI, smoking, alcohol consumption, physical exercise, diabetes, and hypertension was used. All statistical analyses were performed using SAS 9.3 (SAS Institute Inc., Cary, NC, USA) and R programming version 3.4.3 (The R Foundation for Statistical Computing, Vienna, Austria). Results with two-tailed *p*-values less than 0.05 were considered significant.

## 3. Results

### 3.1. Baseline Characteristics

Those who had undergone a medical check-up between 1 January and 31 December 2009 were recruited (*n* = 9,892,633) and were followed up until 31 December 2017. Among the total study population, 5,125,888 individuals were diagnosed with AD, AR, or asthma (AD, 295,466; AR, 4,779,445; and Asthma, 1,184,035).

Table 1 summarizes the initial characteristics of the study group according to the presence or absence of allergic diseases. Individuals with allergic diseases were older and were more likely to be female, have never smoked and abstained from alcohol consumption. The allergy group was less likely to be included in the lowest quintile of income. People with allergic disorders had a higher BMI than those who did not, and were more likely to have diabetes, hypertension, and dyslipidaemia.

### 3.2. Association between Allergic Diseases and the Risk of GI Cancers

In the total study population, the mean follow-up period after a 1-year lag of the diagnosis of allergic diseases was 7.1 ± 1.1 years (control and allergic diseases groups: 7.2 ± 1.0 years and 7.1 ± 1.1 years, respectively).

**Figure 2:**
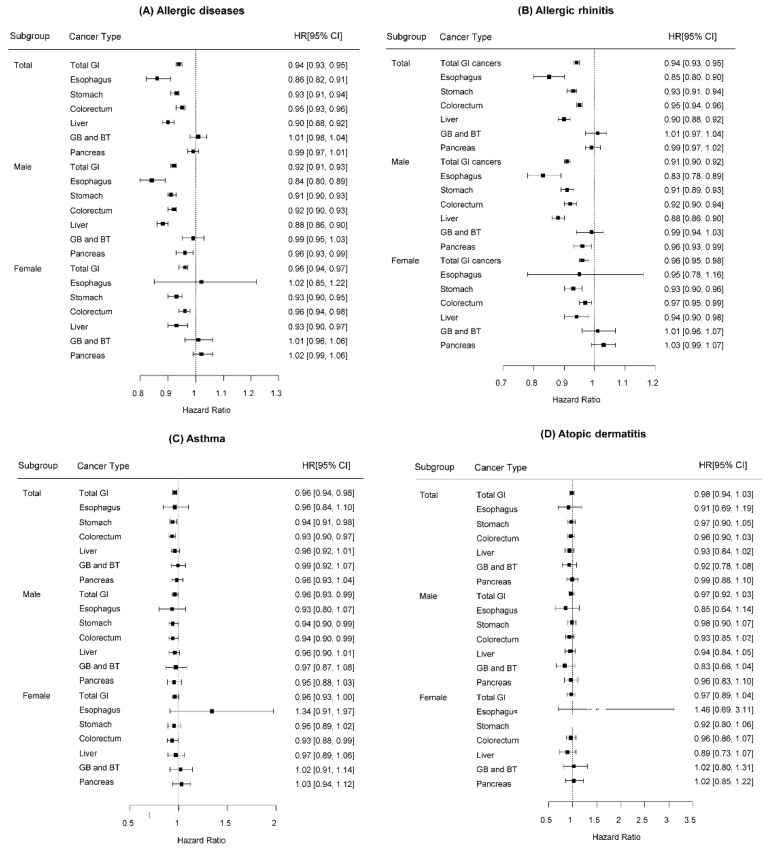
Hazard ratios for the associations of allergic diseases with digestive system cancers. (**A**) allergic diseases, (**B**) allergic rhinitis, (**C**) asthma, (**D**) atopic dermatitis. HR, Hazard ratio; IR, incidence rate; GI, gastrointestinal; GB, gallbladder; BT, biliary tracts.

Allergic diseases were associated with a decrease in the risk of malignancy in the esophagus, stomach, colorectum, and liver, whereas they were not associated with the development of cancers in the pancreas, gallbladder, and biliary tracts (Table 2) (Figure 2A). The aHRs for esophageal, gastric, colorectal, and hepatic cancers were 0.86, 0.93, 0.95, and 0.90 (95% CI 0.82–0.91, 0.91–0.94, 0.93–0.96, and 0.88–0.92), respectively, compared to the controls (Figure 2A).

In sex-specific analyses, the preventive effect of having allergic diseases was persistent on gastric, colorectal, and liver cancers regardless of sex. However, for oesophageal and pancreatic cancers, only males had an inverse association with allergic diseases (esophageal cancers: male 0.84 95% CI 0.80–0.89 vs. female 1.02 95% CI 0.85–1.22; pancreatic cancers: male 0.96 95% CI 0.93–0.99 vs. female 1.02 95% CI 0.99–1.06) (Figure 2A).

Table 3 shows multivariable-adjusted aHRs for the development of GI cancers according to the combination of diagnosed allergic diseases. Compared to healthy controls, having AR was most associated with a decreased risk of neoplasm in the stomach, colorectum, and liver, whereas AD had little association with the neoplasm. As a result, an increase in the number of allergic diseases did not proportionally decrease the risk of cancer.

The coexistence of AR, AD, and asthma reduced the risk of gastric and hepatic cancers by up to 15% (neoplasm of the stomach: HR 0.85, 95% CI 0.77–0.94; liver: HR 0.84, 95% CI 0.74–0.95). Those who had one or more allergic diseases were less likely to develop esophageal cancers; however, patients with all three allergic diseases did not show a significant preventive effect. The risks of cancers on gallbladder and biliary tract or pancreas did not show significant changes in correlation with the number of allergies.

### 3.3. Association between Specific Allergic Diseases and the Risk of GI Cancers

Subgroup analyses were performed after dividing allergic diseases into specific diseases. AR alone was associated with reduced risks for gastric, colorectal, and liver cancers among both men and women and only esophageal and pancreatic cancer among men (Supplementary Table S1) (Figure 2B). Participants with asthma alone showed a reduced risk of stomach and colorectal cancers (Figure 2C). In terms of AD, no association was observed in relation to risk of any GI cancers (Figure 2D). The risks of cancers on the gallbladder and biliary tract were not associated with any of the three allergic diseases (Supplementary Tables S1–S3) (Figure 2).

## 4. Discussion

In this large cohort study, we found that allergic conditions were associated with a decreased risk of gastric, colorectal, and liver cancer among both men and women. As for the oesophageal and pancreatic cancers, only men, not women, were less likely to develop these cancers compared to controls. In a subgroup analysis, according to the specific allergy type, AR alone had the same results as all allergic diseases, while AD did not show associations with any of the GI cancers.

Although several studies have investigated the effect of allergic conditions on malignant diseases, consistent results were not obtained. Moreover, especially in situations where a various relevance with atopic history is expected depending on the cancer location, studies on the relationship between allergic diseases and malignancy in other digestive organs other than colon are rare. In the present study, the inverse association with allergies was persistent in the neoplasms of the stomach, colorectum, and liver, regardless of sex, while pancreatic and esophageal cancers were associated with allergies only in male patients.

Colorectal cancer is one of the most studied carcinomas for its association with allergic diseases. Throughout the literature review, approximately 10–25% of reduction in colorectal cancer risk associated with allergic diseases was reported in three studies [29,30,31]. Among them, no differences emerged according to age, sex, or location of cancers. A meta-analysis, including 515,379 participants from 12 studies showed a 12% reduction in colorectal cancer risk among those who had allergic diseases compared to that among controls [32], which is consistent with our results.

The observed reduction in the risk of colon cancers among atopic individuals may be explained by the immunosurveillance hypothesis [33,34]. According to the theory, it is thought that excessive stimulation of natural killer cells, eosinophils, mast cells, and excessive T-helper cell type II reactions can detect and effectively eliminate damaged cells before the onset of carcinogenesis. In contrast to our result, Ji et al. reported that 140,425 hospitalized patients with asthma had a significant increase in the risk of developing colon cancer [35]. A recently published Taiwanese study also suggested an increased risk of colorectal cancer in patients with atopic dermatitis [36]. However, Ji’s study included more severely ill patients who underwent more imaging and endoscopic work-up, which may have brought different results.

The allergic responses, which start from the airway, may be associated with an increase in leukocytes in the intestinal mucosa of atopic patients [37]. Eosinophils have cytotoxic effects and mediate anti-tumour-like activity in rapidly differentiating tissues, such as intestinal mucosa [38,39]. The level of IgE is elevated in the serum and tissue, indicating the possibility of a systemic effect [40]. An in vitro study reported IgE-induced colon cancer cell apoptosis [41]. In addition, large amounts of lymphocytes are present in the gut-associated lymphoid tissue [42], which might facilitate effective immunosurveillance.

According to the literature, a meta-analysis of 12,712 pooled populations showed that an atopic condition was associated with a 21% decrease in pancreatic cancer risk [15], while Huang et al. revealed no association between allergic conditions and risk of pancreatic cancer [43]. A Taiwanese study had reported a decreased esophageal cancer risk only in male patients with AR, which is consistent with our results [44]. The reason for gender specific results is not clear. This indicates that different pathophysiologies may exist between genders. A further study is required. On the other hand, Finland reported that gastric cancers had an inverse correlation in asthma patients (SIR = 0.88) [45]. Recently, a large case–control study on elderly patients in the United States demonstrated a reduced hazard ratio of esophageal, gastric, colorectal, and liver cancers in patients with AR, which was consistent with our results [46]. However, the US study showed that the risk of developing liver cancer decreased in asthma patients, while those with AD did not show significant association with any GI cancer [46].

The preventive effect was found mainly for infection-related cancers by *Helicobacter pylori*, Hepatitis B, and C viruses or human papillomavirus. *Helicobacter pylori*, *Streptococcus bovis*, JC virus, and human papillomavirus have been evaluated as possible etiologic agents for colorectal cancer [47]. However, in the present study, the inverse association was not seen with gallbladder and biliary tract cancers.

Despite the effort to evaluate the relationship between the type of exposure (AR, AD, or asthma) and cancer risks, the results were inconsistent. In the present study, we observed that atopic dermatitis was not associated with the development of gastric, colorectal cancer, and liver cancers. Atopic dermatitis usually begins in infancy, and many of them tend to cease before adolescence. The nature of this disease course may contribute to null associations between dermatitis and GI cancers in this study for adults. AD, AR, and asthma have similar biological processes of hypersensitivity to allergens, but in the case of AR, inhaled allergens can reach not only the mucous membranes of the airways, but sometimes also the mucosa of the GI tract, which might have different effects on carcinogenesis in GI tracts [48,49]. In many cases, in adults, it is possible that the atopic dermatitis code was incorrectly claimed.

Indeed, the limitation of the operational definition using claim data has been raised. Although we defined allergic diseases using claim data, and not information that relies on self-examination, and added additional conditions, such as three or more claims per year to reduce the inaccuracy of the operational definition, misclassification may exist.

Another limitation is that this study did not include information on anti-allergic drugs that may affect cancer development. The claim data did not contain information on *H. pylori* or hepatitis virus B or C infection.

Despite these limitations, our research has several strengths as it is one of the largest population-based studies on the evaluation of major GI cancer cases.

## 5. Conclusions

In the modern era, where immune checkpoint inhibitor treatments are increasingly applied to solid cancer treatments, we believe that our study showing the reduced risk of most GI cancers in individuals with allergic rhinitis is providing new clues for the treatment of GI cancer. More well-planned mechanistic studies will continue to be published in the future.

## Figures and Tables

**Table 1:** Characteristics of Study Participants at Baseline.

Variables	No Allergic Diseases	Allergic Diseases	*p*
	(*n* = 4,766,745)	(*n* = 5,125,888)	
Sex, Male	2,958,412 (62.1)	2,505,876 (48.9)	<0.0001
Age (years)	46.05 ± 13.82	47.6 ± 14.19	<0.0001
Smoking			<0.0001
Non-smoker	2,581,642 (54.2)	3,266,772 (63.7)	
Ex-smoker	687,730 (14.4)	731,246 (14.3)	
Current-smoker	1,497,373 (31.4)	1,127,870 (22.0)	
Alcohol consumption			<0.0001
Non	2,246,070 (47.1)	2,800,084 (54.6)	
Drinking	2,520,675 (52.9)	2,325,804 (45.4)	
Regular exercise	866,087 (18.2)	954,290 (18.6)	<0.0001
Low income	990,063 (20.8)	1,050,500 (20.5)	<0.0001
BMI (Kg/m^2^)	23.69 ± 3.19	23.73 ± 3.22	<0.0001
Diabetes mellitus	395,243 (8.3)	451,024 (8.8)	<0.0001
Hypertension	1,161,169 (24.4)	1,351,155 (26.4)	<0.0001
Dyslipidemia	796,386 (16.7)	999,591 (19.5)	<0.0001
Atopic dermatitis		295,466 (5.8)	
Allergic rhinitis		4,779,445 (93.2)	
Asthma		1,184,035 (23.1)	
Follow-up duration (years)			
Mean ± SD	7.15 ± 1.04	7.13 ± 1.10	<0.0001
Median (Q1–Q3)	7.3 (7.10–7.57)	7.31 (7.10–7.58)	<0.0001

Values are presented as number (%). BMI, body mass index; SD, standard deviation; Q, quartile.

**Table 2:** Association Between Allergic Diseases and Risk of Gastrointestinal Cancers.

Cancer Type	All	Total			Male			Female		
		*n*	Event	HR ^a^ (95% CI)	*n*	Event	HR ^a^ (95% CI)	*n*	Event	HR ^a^ (95% CI)
Total	No	4,766,745	99,826	1 (Ref)	2,958,412	70,758	1 (Ref)	1,808,333	29,068	1 (Ref)
	Yes	5,125,888	105,124	0.94 (0.93, 0.95)	2,505,876	64,302	0.92 (0.91, 0.93)	2,620,012	40,822	0.96 (0.94, 0.97)
Esophagus	No	4,766,745	3039	1 (Ref)	2,958,412	2834	1 (Ref)	1,808,333	205	1 (Ref)
	Yes	5,125,888	2660	0.86 (0.82, 0.91)	2,505,876	2358	0.84 (0.80, 0.89)	2,620,012	302	1.02 (0.85, 1.22)
Stomach	No	4,766,745	36,254	1 (Ref)	2,958,412	27,283	1 (Ref)	1,808,333	8971	1 (Ref)
	Yes	5,125,888	36,923	0.93 (0.91, 0.94)	2,505,876	24,743	0.91 (0.90, 0.93)	2,620,012	12,180	0.93 (0.90, 0.95)
CRC	No	4,766,745	41,460	1 (Ref)	2,958,412	27,231	1 (Ref)	1,808,333	14,229	1 (Ref)
	Yes	5,125,888	44,938	0.95 (0.93, 0.96)	2,505,876	24,843	0.92 (0.90, 0.93)	2,620,012	20,095	0.96 (0.94, 0.98)
Liver	No	4,766,745	22,801	1 (Ref)	2,958,412	17,671	1 (Ref)	1,808,333	5130	1 (Ref)
	Yes	5,125,888	22,572	0.90 (0.88, 0.92)	2,505,876	15,472	0.88 (0.86, 0.90)	2,620,012	7100	0.93 (0.90, 0.97)
GB/BT	No	4,766,745	7365	1 (Ref)	2,958,412	4595	1 (Ref)	1,808,333	2770	1 (Ref)
	Yes	5,125,888	8970	1.01 (0.98, 1.04)	2,505,876	4842	0.99 (0.95, 1.03)	2,620,012	4128	1.01 (0.96, 1.06)
Pancreas	No	4,766,745	15,921	1 (Ref)	2,958,412	10,746	1 (Ref)	1,808,333	5175	1 (Ref)
	Yes	5,125,888	18,178	0.99 (0.97, 1.01)	2,505,876	10,370	0.96 (0.93, 0.99)	2,620,012	7808	1.02 (0.99, 1.06)

BT, biliary tracts; CI, confidential interval; CRC, colorectum; GB, gallbladder; HR, hazard ratio; IR, incidence rate; N, number; Ref, reference. ^a^ adjusted for age, sex, smoking, alcohol consumption, low income, body mass index, diabetes mellitus, and hypertension.

**Table 3:** The Hazard Ratios of Gastrointestinal Cancers According to Number of Allergic Diseases.

Type	AD/Asthma/AR	*n*	Event	HR (95% CI) ^a^
Esophagus	(−/−/−)	4,766,745	3039	1 (Ref)
	(−/+/−)	246,474	238	0.95 (0.83, 1.09)
	(−/−/+)	3,705,553	1696	0.84 (0.80, 0.90)
	(−/+/+)	878,395	573	0.91 (0.83, 0.99)
	(+/−/−)	91,766	54	0.90 (0.69, 1.18)
	(+/+/−)	8203	10	1.18 (0.63, 2.19)
	(+/−/+)	144,534	58	0.71 (0.55, 0.92)
	(+/+/+)	50,963	31	0.80 (0.56, 1.15)
Stomach	(−/−/−)	4,766,745	36,254	1 (Ref)
	(−/+/−)	246,474	2704	0.95 (0.91, 0.98)
	(−/−/+)	3,705,553	24,534	0.93 (0.91, 0.94)
	(−/+/+)	878,395	7526	0.92 (0.90, 0.94)
	(+/−/−)	91,766	702	0.98 (0.91, 1.05)
	(+/+/−)	8203	86	0.87 (0.71, 1.08)
	(+/−/+)	144,534	951	0.89 (0.84, 0.95)
	(+/+/+)	50,963	420	0.85 (0.77, 0.94)
Colorectum	(−/−/−)	4,766,745	41,460	1 (Ref)
	(−/+/−)	246,474	3139	0.94 (0.91, 0.97)
	(−/−/+)	3,705,553	29,913	0.95 (0.94, 0.97)
	(−/+/+)	878,395	9268	0.94 (0.92, 0.96)
	(+/−/−)	91,766	805	0.96 (0.90, 1.03)
	(+/+/−)	8203	92	0.79 (0.65, 0.97)
	(+/−/+)	144,534	1188	0.94 (0.89, 0.99)
	(+/+/+)	50,963	533	0.91 (0.83, 0.99)
Liver	(−/−/−)	4,766,745	22,801	1 (Ref)
	(−/+/−)	246,474	1739	0.95 (0.91, 1.00)
	(−/−/+)	3,705,553	14,898	0.90 (0.88, 0.92)
	(−/+/+)	878,395	4662	0.90 (0.87, 0.93)
	(+/−/−)	91,766	422	0.93 (0.84, 1.02)
	(+/+/−)	8203	44	0.69 (0.51, 0.93)
	(+/−/+)	144,534	542	0.81 (0.743, 0.88)
	(+/+/+)	50,963	265	0.84 (0.74, 0.95)
Pancreas	(−/−/−)	4,766,745	15,921	1 (Ref)
	(−/+/−)	246,474	1325	0.98 (0.93, 1.04)
	(−/−/+)	3,705,553	11,887	0.99 (0.97, 1.02)
	(−/+/+)	878,395	3881	1.00 (0.96, 1.03)
	(+/−/−)	91,766	322	0.99 (0.88, 1.10)
	(+/+/−)	8203	41	0.87 (0.64, 1.18)
	(+/−/+)	144,534	495	1.01 (0.93, 1.11)
	(+/+/+)	50,963	227	0.96 (0.85, 1.10)
GB/BT	(−/−/−)	4,766,745	7365	1 (Ref)
	(−/+/−)	246,474	719	0.99 (0.92, 1.07)
	(−/−/+)	3,705,553	5662	1.01 (0.97, 1.04)
	(−/+/+)	878,395	2062	1.03 (0.98, 1.08)
	(+/−/−)	91,766	146	0.92 (0.78, 1.08)
	(+/+/−)	8203	28	1.09 (0.75, 1.58)
	(+/−/+)	144,534	235	1.00 (0.87, 1.13)
	(+/+/+)	50,963	118	0.96 (0.80, 1.15)

BT, biliary tracts; CI, confidential interval; GB, gallbladder; GI, gastrointestinal; HR, hazard ratio; IR, incidence rate; No, number; Ref., reference. ^a^ adjusted for age, sex, smoking, alcohol consumption, low income, body mass index, diabetes, and hypertension

## Data Availability

The datasets generated and/or analyzed during the current study are available from the corresponding author upon reasonable request.

## Supplementary Materials

The following supporting information can be downloaded at: https://www.mdpi.com/article/10.3390/cancers15123219/s1, Table S1: Association Between Allergic Rhinitis and Risk of Gastrointestinal Cancers; Table S2: Association Between Asthma and Risk of Gastrointestinal Cancers; Table S3: Association Between Atopic Dermatitis and Risk of Gastrointestinal Cancers.
