# Supplementary material for: Allergic Diseases and Risk of Malignancy of Gastrointestinal Cancers
Source: Cancers (Basel). 2023 Jun 16;15(12):3219. doi: 10.3390/cancers15123219 (PMC10296235; doi:10.3390/cancers15123219)
Supplement: Supplementary file 1 [file cancers-15-03219-s001.zip › cancers-2414825-supplementary.pdf]

Supplementary Table S1. Association between Allergic rhinitis and Risk of Gastrointestinal Cancers

Cancer type	AR	Total				Male				Female			
		n	Event	IR [a]	HR[b] (95% C.I)	*n*	Event	IR [a]	HR[b] (95% C.I)	*n*	Event	IR [a]	HR[b] (95% C.I)
Total GI cancers	No	4766745	99826	2.93	1(Ref.)	2958412	70758	3.35	1(Ref.)	1808333	29068	2.25	1(Ref.)
	Yes	3705553	69481	2.62	0.94 (0.93,0.95)	1880208	43333	3.23	0.91(0.90,0.92)	1825345	26148	2.00	0.96(0.95,0.98)
Esophagus	No	4766745	3039	0.09	1(Ref.)	2958412	2834	0.13	1(Ref.)	1808333	205	0.02	1(Ref.)
	Yes	3705553	1696	0.06	0.85 (0.80,0.90)	1880208	1521	0.11	0.83(0.78,0.89)	1825345	175	0.01	0.95(0.78,1.16)
Stomach	No	4766745	36254	1.05	1(Ref.)	2958412	27283	1.28	1(Ref.)	1808333	8971	0.68	1(Ref.)
	Yes	3705553	24534	0.91	0.93 (0.91,0.94)	1880208	16705	1.23	0.91(0.89,0.93)	1825345	7829	0.59	0.93(0.90,0.96)
Colorectum	No	4766745	41460	1.20	1(Ref.)	2958412	27231	1.27	1(Ref.)	1808333	14229	1.08	1(Ref.)
	Yes	3705553	29913	1.11	0.95 (0.94,0.96)	1880208	16854	1.24	0.92 (0.90,0.94)	1825345	13059	0.98	0.97(0.95,0.99)
Liver	No	4766745	22801	0.66	1(Ref.)	2958412	17671	0.83	1(Ref.)	1808333	5130	0.39	1(Ref.)
	Yes	3705553	14898	0.55	0.90 (0.88,0.92)	1880208	10432	0.76	0.88(0.86,0.90)	1825345	4466	0.33	0.94(0.90,0.98)
GB and BT	No	4766745	7365	0.21	1(Ref.)	2958412	4595	0.21	1(Ref.)	1808333	2770	0.21	1(Ref.)
	Yes	3705553	5662	0.21	1.01 (0.97,1.04)	1880208	3149	0.23	0.99 (0.94,1.03)	1825345	2513	0.19	1.01(0.96,1.07)
Pancreas	No	4766745	15921	0.46	1(Ref.)	2958412	10746	0.50	1(Ref.)	1808333	5175	0.39	1(Ref.)
	Yes	3705553	11887	0.44	0.99 (0.97,1.02)	1880208	6942	0.51	0.96(0.93,0.99)	1825345	4945	0.37	1.03 (0.99,1.07)

AR, Allergic rhinitis; BT, biliary tracts; CI, confidential interval; GB gallbladder; GI, gastrointestinal; HR, hazard ratio; IR, incidence rate; N, number; Ref., reference

[a] per 1000 person-years

[b]Adjusted for age, sex, smoking, alcohol consumption, low income, body mass index, diabetes and hypertension

Supplementary Table S2. Association between Asthma and Risk of Gastrointestinal Cancers

Cancer type	Asthma	Total				Male				Female			
		n	Event	IR [a]	HR[b] (95% C.I)	*n*	Event	IR [a]	HR[b] (95% C.I)	*n*	Event	IR [a]	HR[b] (95% C.I)
Total GI cancers	No	4766745	99826	2.93	1(Ref.)	2958412	70758	3.35	1(Ref.)	1808333	29068	2.25	1(Ref.)
	Yes	246474	7736	4.51	0.96(0.94,0.98)	117344	4787	5.95	0.96(0.93,0.99)	129130	2949	3.23	0.96(0.93,1.00)
Esophagus	No	4766745	3039	0.09	1(Ref.)	2958412	2834	0.13	1(Ref.)	1808333	205	0.02	1(Ref.)
	Yes	246474	238	0.14	0.96(0.84,1.10)	117344	208	0.25	0.93(0.80,1.07)	129130	30	0.03	1.34(0.91,1.97)
Stomach	No	4766745	36254	1.05	1(Ref.)	2958412	27283	1.28	1(Ref.)	1808333	8971	0.68	1(Ref.)
	Yes	246474	2704	1.54	0.94(0.91,0.98)	117344	1815	2.21	0.94(0.90,0.99)	129130	889	0.95	0.95(0.89,1.02)
Colorectum	No	4766745	41460	1.20	1(Ref.)	2958412	27231	1.27	1(Ref.)	1808333	14229	1.08	1(Ref.)
	Yes	246474	3139	1.79	0.93(0.90,0.97)	117344	1799	2.19	0.94(0.90,0.99)	129130	1340	1.44	0.93(0.88,0.99)
Liver	No	4766745	22801	0.66	1(Ref.)	2958412	17671	0.83	1(Ref.)	1808333	5130	0.39	1(Ref.)
	Yes	246474	1739	0.99	0.96(0.92,1.01)	117344	1176	1.42	0.96(0.90,1.01)	129130	563	0.60	0.97(0.89,1.06)
GB and BT	No	4766745	7365	0.21	1(Ref.)	2958412	4595	0.21	1(Ref.)	1808333	2770	0.21	1(Ref.)
	Yes	246474	719	0.41	0.99(0.92,1.07)	117344	375	0.45	0.97(0.87,1.08)	129130	344	0.37	1.02 (0.91,1.14)
Pancreas	No	4766745	15921	0.46	1(Ref.)	2958412	10746	0.50	1(Ref.)	1808333	5175	0.39	1(Ref.)
	Yes	246474	1325	0.75	0.98(0.93,1.04)	117344	740	0.89	0.95(0.88,1.03)	129130	585	0.63	1.03 (0.94,1.12)

BT, biliary tracts; CI, confidential interval; GB gallbladder; GI, gastrointestinal; HR, hazard ratio; IR, incidence rate; N, number; Ref., reference

[a] per 1000 person-years

[b]Adjusted for age, sex, smoking, alcohol consumption, low income, body mass index, diabetes and hypertension

Supplementary Table S3. Association between Atopic dermatitis and Risk of Gastrointestinal Cancers

Cancer type	AD	Total				Male				Female			
		n	Event	IR [a]	HR[b] (95% C.I)	*n*	Event	IR [a]	HR[b] (95% C.I)	*n*	Event	IR [a]	HR[b] (95% C.I)
Total GI cancers	No	4766745	99826	2.93	1(Ref.)	2958412	70758	3.35	1(Ref.)	1808333	29068	2.25	1(Ref.)
	Yes	91766	1965	3.00	0.98(0.94,1.03)	46775	1308	3.94	0.97(0.92,1.03)	44991	657	2.04	0.97(0.89,1.04)
Esophagus	No	4766745	3039	0.09	1(Ref.)	2958412	2834	0.13	1(Ref.)	1808333	205	0.02	1(Ref.)
	Yes	91766	54	0.08	0.91(0.69,1.19)	46775	47	0.14	0.85(0.64,1.14)	44991	7	0.02	1.46(0.69,3.11)
Stomach	No	4766745	36254	1.05	1(Ref.)	2958412	27283	1.28	1(Ref.)	1808333	8971	0.68	1(Ref.)
	Yes	91766	702	1.06	0.97(0.90,1.05)	46775	508	1.51	0.98(0.90,1.07)	44991	194	0.59	0.92(0.80,1.06)
Colorectum	No	4766745	41460	1.20	1(Ref.)	2958412	27231	1.27	1(Ref.)	1808333	14229	1.08	1(Ref.)
	Yes	91766	805	1.21	0.96(0.90,1.03)	46775	485	1.44	0.93(0.85,1.02)	44991	320	0.98	0.96(0.86,1.07)
Liver	No	4766745	22801	0.66	1(Ref.)	2958412	17671	0.83	1(Ref.)	1808333	5130	0.39	1(Ref.)
	Yes	91766	422	0.63	0.93(0.84,1.02)	46775	315	0.93	0.94(0.84,1.05)	44991	107	0.33	0.89(0.73,1.07)
GB and BT	No	4766745	7365	0.21	1(Ref.)	2958412	4595	0.21	1(Ref.)	1808333	2770	0.21	1(Ref.)
	Yes	91766	146	0.22	0.92(0.78,1.08)	46775	79	0.23	0.83(0.66,1.04)	44991	67	0.20	1.02(0.80,1.31)
Pancreas	No	4766745	15921	0.46	1(Ref.)	2958412	10746	0.50	1(Ref.)	1808333	5175	0.39	1(Ref.)
	Yes	91766	322	0.48	0.99(0.88,1.10)	46775	198	0.59	0.96(0.83,1.10)	44991	124	0.38	1.02(0.85,1.22)

AD, atopic dermatitis; BT, biliary tracts; CI, confidential interval; GB gallbladder; GI, gastrointestinal; HR, hazard ratio; IR, incidence rate; N, number; Ref., reference

[a] per 1000 person-years

[b]Adjusted for age, sex, smoking, alcohol consumption, low income, body mass index, diabetes and hypertension
